# Charting New Territory With a Novel WHO Provisional Category: A Case Study of a Low-Grade Oncocytic Tumor (LOT) in the Kidney

**DOI:** 10.7759/cureus.78132

**Published:** 2025-01-28

**Authors:** Jayalakshmi N Alagar, Kassaye Firde, Binyam Fentaw, Samir M Amer, Anjali Seth, Daniela Proca

**Affiliations:** 1 Pathology and Laboratory Medicine, Temple University Hospital, Philadelphia, USA; 2 Molecular Pathology, Temple University Hospital, Philadelphia, USA; 3 Pathology, Temple University, Philadephia, USA

**Keywords:** chromophobe renal carcinoma, immunohistochemistry, low-grade oncocytic tumors of the kidney, renal, renal oncocytoma

## Abstract

A low-grade oncocytic tumor of the kidney (LOT) is a distinctive entity categorized under the 2022 World Health Organization (WHO) classification as "Other oncocytic tumors of the kidney.” It represents a unique subset of oncocytic tumors, distinct from oncocytoma and chromophobe renal cell carcinoma. Characterized by bland oncocytic cells that diffusely express CK7 and lack CD117 expression, LOT typically manifests as a solitary, small lesion with a low stage. Available data suggest that LOT follows an indolent clinical course. Herein, we present a case of LOT in a 64-year-old male with a favorable course at a one-year follow-up. Understanding the clinical, morphological, and immunophenotypic features of LOT is paramount for accurate identification and characterization. This knowledge facilitates more conservative surgical and clinical management, optimizing patient care and outcomes.

## Introduction

As the most common finding in renal neoplasia in pathology, eosinophilic cytoplasm is characteristic of multiple renal tumors [1]. High on the differential diagnosis are two well-recognized, non-papillary renal neoplasms: renal oncocytoma (RO) and the eosinophilic variant of chromophobe renal cell carcinoma (eo-ChRCC). Distinguishing between these entities is necessary for proper tumor management since RO is benign, whereas eo-ChRCC, which accounts for 5% of all malignant renal neoplasms, has metastatic potential [2]. Both tumors have distinctive morphological and immunohistochemical profiles that aid in diagnosis during the initial workup [3]. RO often has a CD117+/ minimal CK7+ (clusters less than 5% of tumor) immunophenotype, uniform nuclei with minimal atypia, mitochondrial gene mutations, and recurrent chromosomal losses (1, 14, 21, X, and Y). CD117+/CK7+ (diffuse, uniform), as well as E-cadherin and N-cadherin + reactivity, raisinoid nuclei of varied size with “halo” surroundings, and multiple, recurrent chromosomal losses (e.g. 1, 2, 6, 10, 13, and 17) that are more suggestive of eo-ChRCC [4,5]. Vimentin and carbonic anhydrase IX (CA-IX) are generally negative in RO and eo-ChRCC [4-6].

The morphological spectrum of oncocytic tumors has widened over the past decades, driven by reports of tumors exhibiting either “mixed” features of both RO and eo-ChRCC together or “borderline” features in between the two [3]. To reflect this increased diversity, the fifth edition of the WHO classification (2022) of urinary and male genital tumors introduced the category “oncocytic and chromophobe renal tumors”, which clearly specifies tumors as RO, ChRCC, or “other oncocytic tumors of the kidney.” The latter subgroup encompasses “low-grade oncocytic tumor” (LOT), “eosinophilic vacuolated tumor” (EVT), “hybrid oncocytic/chromophobe tumors” (HOCT), and a heterogeneous group of sporadic eosinophilic/oncocytic tumors with borderline features that await further classification [6].

LOT, first described by Trpkov and Hes in 2019, is now recognized as an emerging entity by the WHO. LOT typically presents as a solitary, low-stage lesion with an indolent behavior [4]. Morphologically, LOT features eosinophilic cytoplasm, bland nuclei with round to oval morphology, and occasional focal perinuclear halos, resembling both RO and eo-ChRCC. However, LOT is distinguished by an edematous stroma, sometimes described as a “boats in a bay” appearance, as well as its immunohistochemical profile: CD117-/CK7+ (diffuse) and GATA3 expression [6,7].

Under-recognition of LOT has historically resulted in misdiagnoses, underscoring the need for further studies to better characterize its features and behavior [6,8]. Accurate identification of LOT is essential to avoid overtreatment and to improve surgical and clinical decision-making for low-risk renal tumors. This case report contributes to the growing body of literature on LOT, supporting the refinement of diagnostic criteria and ensuring optimal patient outcomes.

## Case presentation

**Figure 1:**
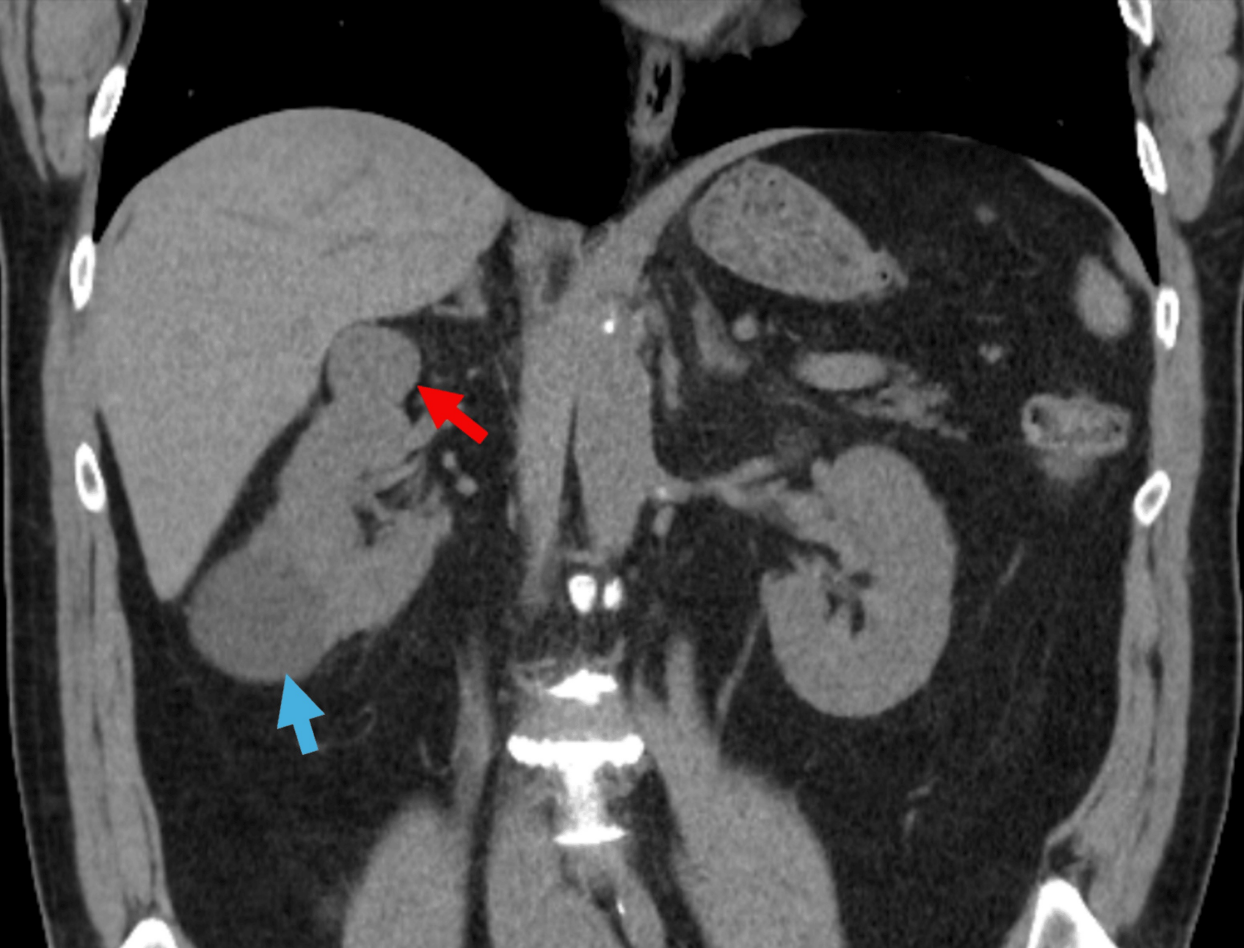
CT abdomen (coronal view) demonstrating a 3.1 cm, enhancing, right upper pole renal mass (red arrow) and a 5.2 cm, large, lower pole simple cyst (blue arrow)

**Figure 2:**
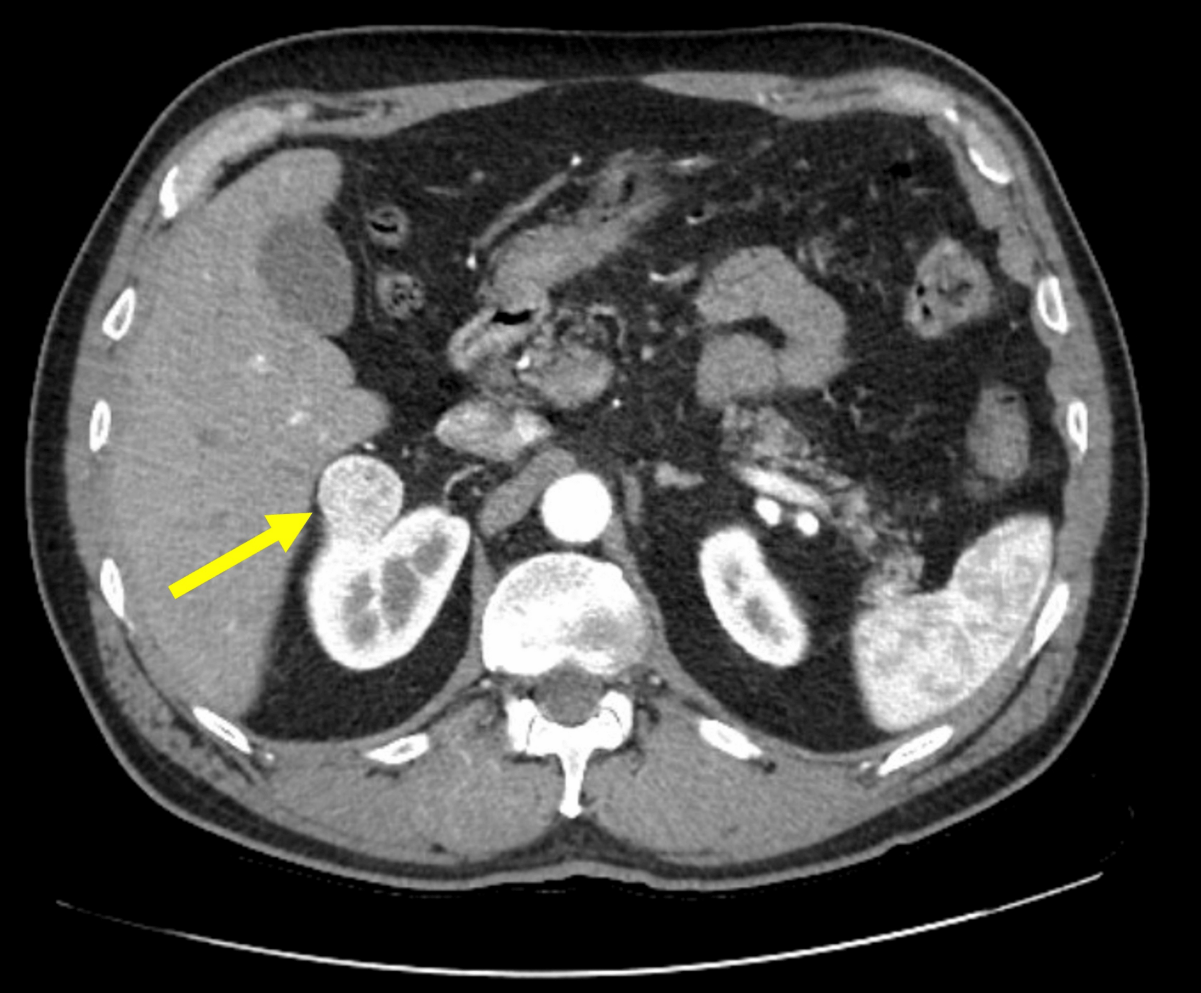
CT abdomen (axial view) demonstrating a 3.1 cm mass in the upper pole of the right kidney (marked by yellow arrow).

The patient is a 64-year-old male presented with recurrent flank pain with renal stones. A CT of the abdomen and pelvis revealed a 3.1 x 2.9 x 2.3 cm partially exophytic hypervascular mass within the anterior aspect of the right renal upper pole that abuts the posterior right hepatic lobe/caudate and a 5.2 x 6.4 X 5.5 cm exophytic simple cyst within the posterior aspect of the inferior right renal pole (Figure 1 and Figure 2). He underwent a right robotic-assisted partial nephrectomy and right renal cyst decortication. The partial nephrectomy specimen was submitted to pathology for gross and microscopic examination. Gross findings revealed a well-circumscribed, tan, soft mass measuring 2.0 x 1.7 x 1.5 cm. The tumor was limited to the kidney and showed no invasion through the capsule or extension into the perinephric fat. The renal parenchymal margin was negative for the tumor.

**Figure 3:**
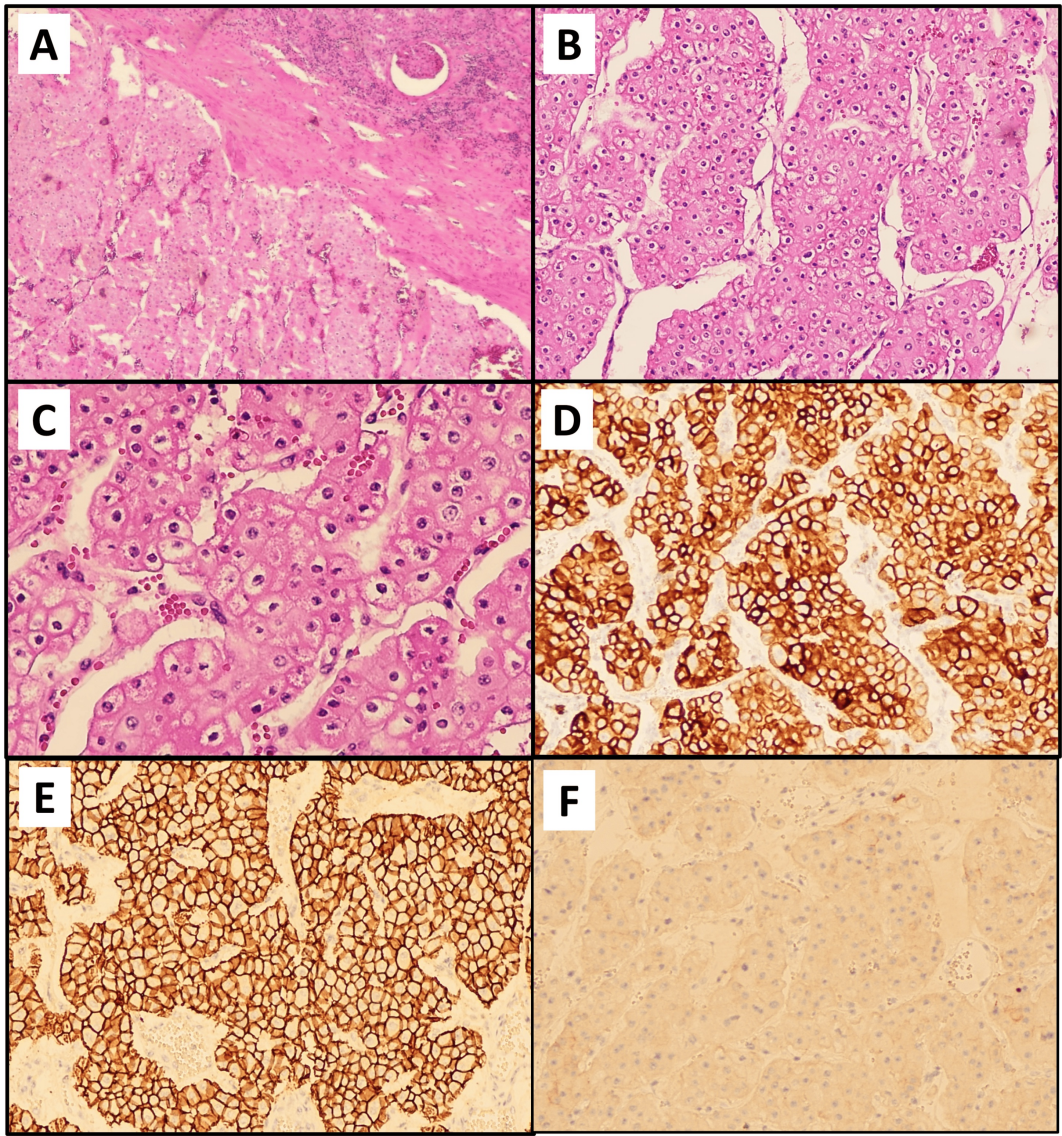
Microscopic examination of LOT: A) The tumor irregularly interfaced with the adjacent renal parenchyma and lacked a capsule (H&E, 4x); B) tumor with oncocytoma-like cytology (H&E, 10X); C) On high power, many oncocytic cells showed perinuclear halos with a focal infiltrate of lymphocytes (H&E, 20X); D) tumor cells show diffuse CK7 positivity (10X); E) tumor cells with diffuse E-cadherin positivity (10X); F) tumor cells showed negative staining for c-kit (10X) LOT: low-grade oncocytic tumor; H&E: hematoxylin and eosin

With hematoxylin and eosin staining, the tumor showed sheets, cords, and a nest of bland eosinophilic cells with ISUP/WHO nuclear grade 1/3 and very rare mitoses. No necrosis, atypical mitoses, significant pleomorphism, or papillary structures were identified upon thorough sampling and examination (Figure 3, panels A, B, C).

Immunohistochemistry revealed the tumor cells were diffusely and strongly positive for both CK7 and E-cadherin (two blocks stained), positive for PAX-8 (nuclear staining), and negative for c-kit, vimentin, DOG-1, progesterone receptor (PR), carbonic anhydrase IX (Figure 3, panels D, E, F). Rare cells showed weak positive staining with racemase and Hale colloidal iron. Ki-67 showed less than 3% nuclear staining. RNA and DNA next-generation sequencing (NGS) testing was negative. The final diagnosis was a low-grade oncocytic tumor (LOT). At the 12-month follow-up, the patient was alive and well, with no evidence of recurrence or metastases. Given the relatively indolent pathology, the follow-up plan included alternating ultrasound and cross-sectional imaging every year for the next few years.

## Discussion

The list of renal neoplasms with eosinophilic cytoplasm is continuously growing. Eosinophilic cytoplasm is a well-known hallmark of oncocytic tumors, where it reflects the cellular accumulation of altered mitochondria during a state of metaplasia that ultimately leads to both neoplastic and non-neoplastic “oncocytic” changes [9]. For pathologists, differentiating oncocytic tumors with marked eosinophilia poses a significant challenge, as different eosinophilic tumors often share multiple, additional features on histopathology. Consequently, it is not uncommon to confuse these benign tumors with malignant mimickers, including eo-ChRCC, an eosinophilic variant of clear cell renal cell carcinoma (eo-RCC), succinate dehydrogenase deficient RCC, hybrid oncocytic chromophobe tumor (HOCT), and fumarate hydratase deficient RCC with low-grade features [10].

However, it is important to correctly identify LOTs of the kidney because of their distinct clinical behavior and the implications for patient management. LOTs are generally indolent, with a very low risk of progression or metastasis, unlike other oncocytic renal tumors, such as the eo-RCC or eo-ChRCC. Misdiagnosis can lead to unnecessarily aggressive treatment, such as radical nephrectomy, which could be avoided with an accurate diagnosis [2]. Proper identification of LOT ensures patients are spared undue anxiety and invasive procedures, allowing for more conservative management when appropriate. Large-scale retrospective studies have indicated that LOT would have been the proper diagnosis for ~5% of cases initially labeled as either RO, ChRCC, or “unclassified RCC or low-grade oncocytic/eosinophilic renal neoplasms” [11,12]. There are no studies on long-term follow-up in LOT patients, but we believe the absence of disease progression of any type in all reported cases so far argues against overtreatment as a standard of care [11].

LOTs must be carefully differentiated from other oncocytic tumors, including RO, ChRCC, eo-RCC, and hybrid tumors often associated with Birt-Hogg-Dubé syndrome [12]. Morphologically, LOTs display a low-grade appearance with uniform oncocytic cells, round-oval nuclei, and smooth nuclear contours, lacking the significant atypia seen in eo-ChRCC and eo-RCC. Unlike classic RO, which is often well-circumscribed, LOTs exhibit a solid, nested architecture. Furthermore, LOTs lack the plant-cell-like morphology and prominent perinuclear halos commonly observed in eo-ChRCC [6,7].

Immunohistochemistry (IHC) is a critical tool in differentiating LOTs from other tumors. LOTs are diffusely positive for CK7, but negative with CD117, distinguishing them from RO and eo-ChRCC. In addition, LOTs are diffusely positive with GATA-3 and E-cadherin, and negative for vimentin, which helps separate them from eo-RCC and eo-ChRCC. Meanwhile, RO is strongly positive for CD117, but generally negative for CK7 (rare positive cells or minute clusters of positive cells may be seen, but represent less than 5% of the tumor) [12-14]. These immunophenotypic features are key in narrowing down the diagnosis.

A chromosomal analysis provides further differentiation, as LOT generally exhibits a diploid stable karyotype with minimal chromosomal alterations, while the most common reported mutations are those affecting the mTOR pathway (MTOR), tuberous sclerosis complex-1 (TSC1), and TSC2 [12]. In contrast, ChRCC often demonstrates widespread chromosomal losses and clear cell RCC is characterized by VHL gene mutations [13,14]. By combining morphological evaluation, immunohistochemical findings, and molecular profiling, pathologists can confidently diagnose LOTs and distinguish them from other renal oncocytic neoplasms [13,14].

The clinicopathological features of LOTs in our patient align well with those previously described. LOTs have been reported in patients aged 10 to 87 years (mean: 62.7) as incidental findings on imaging in all poles of the kidney. They exhibit a slight preference for females and typically measure up to 3-4 cm in diameter. LOTs are usually sporadic, single tumors in non-syndromic settings, although multiple and/or bilateral LOTs have been observed in association with TSC and end-stage renal disease [15].

Studies have consistently shown that the overall incidence rate of LOT among all renal tumors is low (<1%) [15]. This could reflect the tumor’s natural rarity and/or the difficulty in recognizing LOT due to its tendency to progress asymptomatically, behave indolently, and share morphological characteristics with other tumors [15].

In summary, awareness and accurate diagnosis of LOTs are crucial for preventing overtreatment and ensuring appropriate patient care. While LOTs share overlapping features with other oncocytic tumors, their indolent clinical behavior and specific histological, immunohistochemical, and molecular characteristics allow for their precise identification.

## Conclusions

With approximately 100 reported cases in the literature, the incidence of LOTs among all renal epithelial tumors is estimated at a mere 0.35%. Remarkably, LOT has an indolent prognosis, with no reported cases of recurrences or metastases. It is imperative to be aware of this distinct entity and its characteristic phenotype to avoid misdiagnosis, particularly with eo-ChRCC and eo-RCC, especially when relying solely on CK7 in the diagnostic assessment of such tumors.
